# Barriers and Facilitators to COVID19 Vaccination Access Among Older Adults: A Comprehensive Needs Assessment for Vaccination Strategies

**DOI:** 10.1017/ash.2025.302

**Published:** 2025-09-24

**Authors:** Jiye (Cecilia) Lee, Jamie Trumpler, Scott Roberts, Lauren Pischel, Richard Martinello

**Affiliations:** 1 Yale University; 2 Yale University; 3 Yale; 4 Yale University

## Abstract

**Objectives:** Multiple barriers exist for COVID-19 vaccination in high-risk individuals especially adults over the age of 65. Each healthcare visit represents a critical opportunity for vaccination, yet many patients who do seek vaccination receive their vaccines in locations other than their routine health care providers and healthcare sites often lack the capacity for vaccine administration. Here-in we conducted a needs assessment to identify hospital system specific barriers and facilitators to COVID-19 vaccine access in individuals 65 and older in 2024. **Methods:** We conducted six semi-structured interviews (June-July 2024) with seven healthcare leaders in Yale New Haven Enterprise. We transcribed and analyzed interviews to develop a larger-scale survey targeting healthcare professionals including vaccine leadership of individual clinics across the healthcare systems. The survey was distributed to 42 healthcare leaders (physicians, administrators, and practice supervisors) across 52 ambulatory locations. **Results:** The survey received twenty responses (47% response rate). Four primary challenges to COVID-19 vaccination among older adults were identified: (1) Patient Hesitancy, driven by misinformation about vaccine contents, concerns about side effects, polarized attitudes, and waning interest in booster doses; (2) Challenges Related to Staff, including distrust in vaccine motives, mandates, and efficacy, as well as a shortage of personnel available to administer vaccinations; (3) Operational and Logistical Barriers, including complex vaccine schedules, vaccine storage, and reliance on retail pharmacies, which led to lower vaccination rates at primary care sites; and (4) Policy and Financial Constraints, such as insufficient financial incentives for on-site vaccinations, Medicare coverage limitations, and high administrative costs. The main proposed actions to address vaccination hesitancy and challenges include enhancing education sessions for patients and staffs, modifying streamlining administration by simplifying the workflows including on-site vaccination process for employees, and centralizing vaccine delivery in primary care or hubs, improving accessibility via routine (home) visits and flexible hours, and partnering with pharmacy department to ensure greater access to vaccination. **Conclusion:** Through semi-structured interviews and surveys, we identified targets for future quality improvement efforts. Multiple overlapping barriers to COVID-19 vaccination in older adults exist within one U.S. based health care system. Some of these barriers, such as improving vaccine administration workflows or enhancing patient education, can be more readily addressed, while others involve larger structural issues that would require larger societal change. We are seeking a subspecialty clinic partnership to pilot an implementation project, using high-impact intervention tailored to clinic needs and iterative Plan-Do-Study-Act (PDSA) cycles to refine and optimize outcomes.

